# Neuronal Control of Posture in Blind Individuals

**DOI:** 10.1007/s10548-024-01041-7

**Published:** 2024-03-15

**Authors:** I. Helmich, R. Gemmerich

**Affiliations:** 1 Department of Sport Science, University of Goettingen, Goettingen, Germany; 2 Department of Motor Behavior in Sports, German Sport University Cologne, Am Sportpark Müngersdorf 6, 50933 Cologne, Germany

**Keywords:** Blindness, Neuroplasticity, Postural control, fNIRS, Motor-sensory cortex

## Abstract

The control of posture is guided by the integration of sensory information. Because blind individuals cannot apply visual information to control posture as sighted individuals do they must compensate by the remaining senses. We therefore hypothesize that blind individuals alter their brain activation in the sensorimotor cortex during postural control to compensate for balance control without vision by the increased integration of somatosensory information. Ten blind and ten sighted (matched) individuals controlled posture during conditions with (I) eyes closed / open, and (II) stable / unstable surface conditions. Postural sway was recorded by applying a pressure distribution measuring plate. Brain activation was collected by functional Near InfraRed Spectroscopy (fNIRS) above motor-sensory cortices of the right and left hemispheres. Blind individuals showed significantly increased postural sway when balancing with open eyes on an unstable surface and when compared to sighted individuals. Whereas blind individuals showed significantly increased brain activation when balancing with open eyes on stable and unstable surface conditions, sighted individuals increased their brain oxygenation only during closed eyes and unstable surface conditions. Overall conditions, blind individuals presented significantly increased brain activation in two channels of the left and right hemispheric motor-sensory cortex when compared to sighted individuals. We therefore conclude that sighted individuals increase their brain oxygenation in the sensorimotor cortex during postural control tasks that demand sensory integration processes. Blind individuals are characterized by increased brain activation overall conditions indicating additional sensory integration during postural control. Thus, the sensorimotor cortex of blind individuals adapts to control posture without vision.

**Supplementary Information:**

The online version contains supplementary material available at 10.1007/s10548-024-01041-7.

## Introduction

Postural control concerns the human ability to keep the body in balance. Impaired postural control is related to an increased risk of fall-related injuries (Johansson et al. 2017), which constitute a major health concern causing functional decline, increased mortality, as well as increased healthcare costs (Wolinsky et al. 1997; Stel et al. 2004). The aim of postural control constitutes postural orientation and postural equilibrium, which involves the active alignment of the human body with respect to gravity, support surfaces, the visual surround and internal references (Horak 2006). Therefore, sensory information from somatosensory, vestibular and visual systems is integrated for postural control.

It has been shown that sight plays a significant role in the control of posture. The absence of sight in blind individuals leads to impaired postural balance (Choy et al. 2003), which results in higher instability in static and dynamic balance tasks (Ribadi et al. 1987; Rougier and Farenc 2000; Nakata and Yabe 2001; Aydoǧ et al. 2006; Giagazoglou et al. 2009; Schwesig et al. 2011; Ozdemir et al. 2013; Sobry et al. 2014). Sobry et al. (2014) compared the static balance control of visually impaired individuals with sighted individuals during conditions of hard (/stable) or soft (/unstable) surfaces and as well as during open and closed eyes conditions. The authors found that visually impaired individuals present higher speeds of displacement on hard surfaces than control subjects. During unstable surface conditions, the speed was higher in visually impaired individuals with open eyes whereas no differences were found with closed eyes (Sobry et al. 2014).

The investigation of the strength of the knee and ankle muscles and its relation to postural sway in blind and sighted women showed that blind women are generally more unstable during static balance tasks such as normal stance, one-leg stance, and/or tandem stance (Giagazoglou et al. 2009). However, no differences were found for the strength measurements of the lower limb muscles between the blind and sighted individuals. Nakata and Yabe (2001) investigated postural sway after perturbation in congenitally blind and sighted adults. The authors also recorded EMG signals from four muscles in the right leg, and reaction time to somatosensory stimuli generated by platform displacements by pushing a hand-held button. No significant differences were found for postural sway between blind and sighted subjects with eyes open or closed. However, blind subjects swayed more after backward translations than sighted subjects with open eyes. The EMG amplitude in the gastrocnemius muscle of a blind subject was smaller than that of a sighted subject with eyes closed. No significant differences were found between blind and sighted subjects in EMG latencies. However, blind individuals were characterized by faster reaction times to somatosensory stimuli triggered by platform displacements. The authors assumed that blindness may not affect the spinal stretch reflex, but may affect a volitional act mediated through the motor cortex (Nakata and Yabe 2001). This assumption is in line with the hypothesis that congenitally blind individuals may adapt to control balance without vision because they showed to be more stable during balance tasks when compared to sighted but blindfolded individuals (Ribadi et al. 1987). In fact, Schwesig et al. (2011) concluded that the somatosensory and vestibular systems may serve as compensatory mechanisms, which is utilized most effectively by the congenitally blind individuals.

The presented findings indicate that blind individuals may adapt to control of balance without vision. Because blind individuals are faced with the fact that visual and multisensory brain regions do not receive their expected input they are forced to adapt to the environment by using their remaining senses to cope with everyday demands. Neuroplasticity has been described as a key characteristic of blind individuals with regard to their adaptation to the environment without vision (Roeder et al. 2021). For example, when attending to sounds in peripheral auditory space blind participants display superior localization abilities when compared to sighted individuals (Röder et al. 1999). Additional electrophysiological recordings revealed sharper tuning of early spatial attention mechanisms in the blind subjects (Röder et al. 1999). The long-term concentration of blind individuals on nonvisual cues to interact appropriately with the environment further showed to enlarge the auditory cortex by a factor of 1.8 when compared to sighted individuals (Elbert et al. 2002). When controlling posture blind individuals must increase their attention to nonvisual sensory cues such as proprioception. This may alter the neuronal control of balance in the sensorimotor cortex of blind individuals.

The study of brain function during postural control in humans is difficult to investigate because standard neuroimaging techniques such as for example functional magnetic resonance imaging (fMRI) do not allow for investigations in upright (/standing) positions. Functional near-infrared spectroscopy (fNIRS) enables the study of neuronal processes (Scholkmann et al. 2014) with the particular advantage of recording of brain activation during the execution of movements in a near-natural context such as during upright/standing positions (Mihara et al. 2008; Karim et al. 2012, 2013; Basso Moro et al. 2014; Ferrari et al. 2014; Helmich et al. 2016, 2020; Herold et al. 2017; Lin et al. 2017; Teo et al. 2018; Lehmann et al. 2022). Studies that applied fNIRS to investigate the neuronal control of balance have shown that frontal, temporal, parietal, and occipital regions are involved in visual and somatosensory integration (Ferrari et al. 2014; Helmich et al. 2016, 2020, 2022; Lin et al. 2017; Mihara et al. 2008). When visual input was not available during postural control, brain oxygenation increased in frontal, temporal, and parietal regions as a process of sensory re-weighting (Lin et al. 2017). This indicates that blind individuals may be characterized by increased brain oxygenation when compared to sighted individuals in sensorimotor cortex during the control of posture, particularly when comparing the two groups during balance conditions with open eyes.

## Materials and Methods

### Participants

An a priori power analysis by using G*Power 3.1.9.7 indicated that 20 participants are necessary for a statistical analysis between groups and repeated measures (effect size f = 0.35, Power = 0.95, calculated critical F value = 2.775, calculated actual Power = 0.96). 20 individuals (mean age: 27.0 ± 6.1 years; 3 female, 17 male; 16 right-handed, 3 left-handed, 1 ambidextrous; years at school: 13 years) therefore participated in the study (Table 1). Participants were divided in two groups: 10 blind individuals (blindness according to the German law; mean age: 29.0 ± 7.1 years; 2 female, 8 male; 9 right-handed, 1 left-handed) and 10 sighted individuals (mean age: 25.0 ± 4.4 years; 1 female, 9 male; 7 right-handed, 2 left-handed, 1 ambidextrous; the control group was matched with the blind group with regards to age, i.e., there was no significant age difference between group (t(18) = 1.524, p = 0.145)). Blindness according to the German law considers individuals who have no sight at all or who have a visual acuity of no more than 0.02 (1/50) in the better eye or with both eyes. Blindness occurred mostly due to Stargardt disease, glaucoma, and/or cataracts. Six blind individuals were blind from birth (congenital) and four participants acquired blindness during life (late blind; Table 1). Average years of blindness in late blind individuals constituted 14.5 ± 5.3 years. All participants were active athletes from nearby sport clubs and did not have any neurological or psychiatric disorder. The local Ethics Committee of the German Sport University approved the study (Nr. 182/2020).

**Table 1:** Participants

ID	Blindness*	Age	Years of blindness / age of onset	Gender	Residual light perception	Years at school	Cause of blindness
01	late blind	43	14 / 29	male	yes	12	Morbus Stargradt
02	late blind	30	10 / 20	male	yes	12	Leber hereditary optic neuropathy (LHON)
03	congenital blind	38		male	yes	12	glaucoma, cataract
04	congenital blind	23		female	yes	12	glaucoma, cataract
05	congenital blind	31		male	yes	12	glaucoma, cataract
06	late blind	25	22 / 3	male	no	12	Iris inflammation
07	late blind	22	12 / 10	male	no	12	glaucoma
08	congenital blind	24		male	no	12	-no information-
09	congenital blind	23		male	no	12	glaucoma
10	congenital blind	31		female	yes	12	glaucoma, cataract
11	sighted	30		male		12	
12	sighted	22		male		12	
13	sighted	20		male		12	
14	sighted	24		male		12	
15	sighted	24		male		12	
16	sighted	22		female		12	
17	sighted	30		male		12	
18	sighted	23		male		12	
19	sighted	22		male		12	
20	sighted	33		male		12	

^*^blindness according to the German law (congenital or late blind)

## Experimental Setting and Study Design

### Posturography, Balance Tasks and Data Collection

**Fig. 1:**
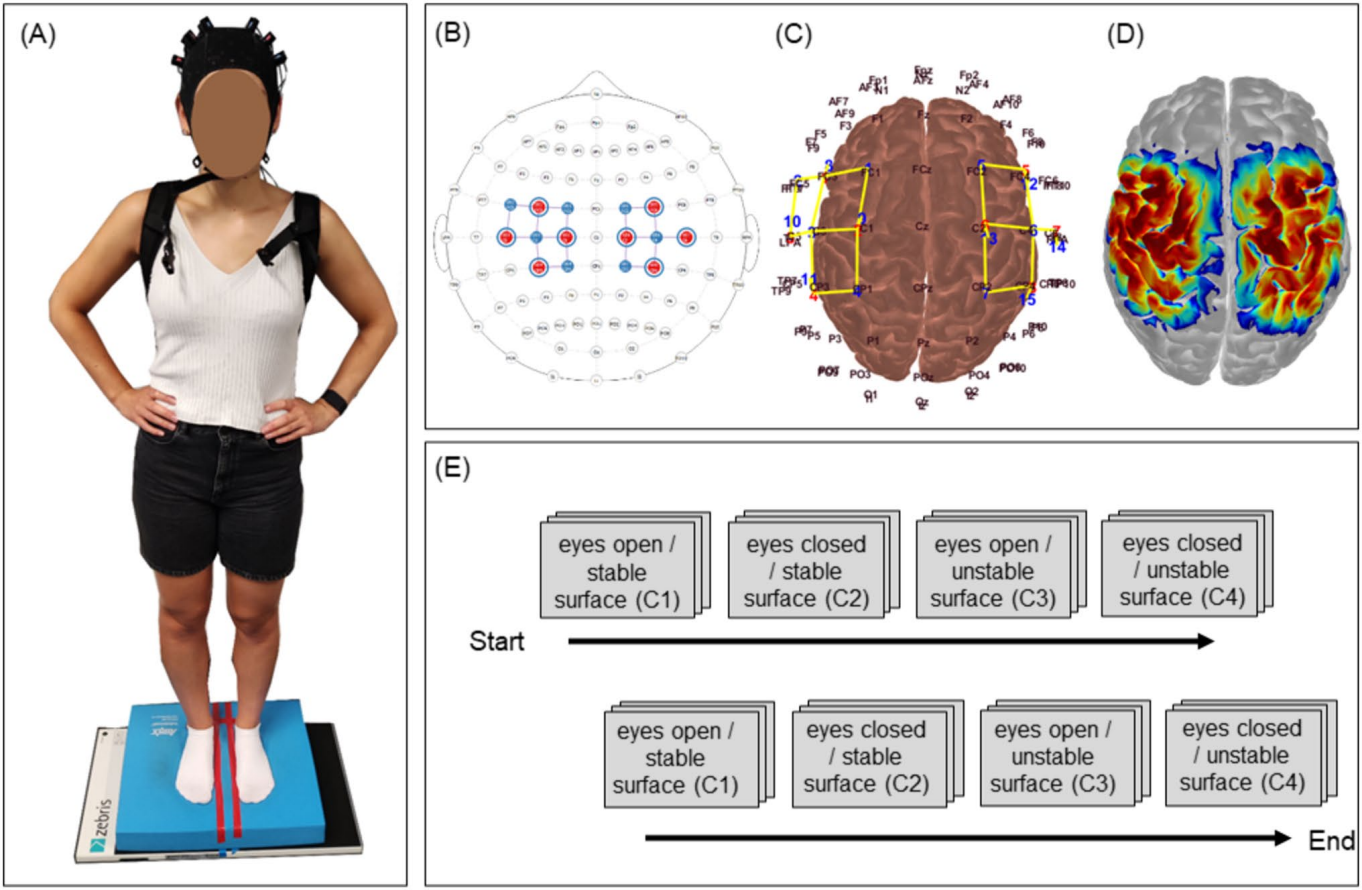
**A** Exemplary measurement/participant; **B** fNIRS optode placement according to the 10–20-system; **C** fNIRS topographical layout; **D** fNIRS sensitivity map; **E** block design (6 trials per condition separated in 2 blocks)

Four postural control conditions were carried out according to Shumway-Cook and Horak (Shumway-Cook and Horak, 1986), which examine combinations of visual and tactile manipulations during balance control: condition 1 (c1): *eyes opened*; condition 2 (c2): *eyes closed*. The two conditions (c1 and c2) were performed either on a firm (/*stable*) surface or on an *unstable* surface: condition 3 (c3): *eyes opened and unstable surface*; condition 4 (c4): *eyes closed and unstable surface*. The unstable surface was created using a piece of six cm thick foam pad ("AIREX Balance-Pad “). Each balance condition comprised two blocks, each of which included three trials (ten seconds [s] per trial; 15 s between conditions / instruction; Fig. 1), resulting in a total of six trials per condition. The subjects were instructed to stand still on both feet without losing balance in a standardized position (distance between feet: 2 cm) and posture (Fig. 1). During postural control tasks, a force plate system („ZEBRIS platform, type FDM-S”, measure frequency 240 Hz) was used to register center of mass displacement (/postural sway) by measuring ground reaction forces. This system provides information about the ability to keep postural control by the registration of the deviations from the Center of Pressure (CoP) by the mean length of the movement path per time (*millimeters/second [mm/s]*); COP *length* is defined as the absolute length of the CoP path movements throughout the testing period (10 s). The second parameter COP *surface [mm*^*2*^*]* provides information about the area used for balancing during the ten seconds. The means of COP *length* and *surface* were exported for each subject and condition for statistical analyses. Because both parameters revealed similar results we focussed in the results section on the COP *length* parameters only.

### fNIRS Acquisition and Analysis

Cerebral oxygenation changes were recorded using an portable continuous wave fNIRS system (NIRSport 2, NIRx, Medical Technologies LLC, Berlin, Germany; wavelengths of 760 nm and 850 nm; sampling rate 10,2 Hz). The system contains eight light sources and eight light detectors, and a short-distance detector bundle. The optodes were placed according to the 10–20-system (Jasper, 1958) above pre- and postcentral gyri of each hemisphere (Table 2; Fig. 1) using a standardized cap (EasyCap GmbH, Herrsching, Germany). Data was recorded from 18 (long-distance) channels of measurement and eight short-distance (8 mm) channels to account for changes in extra-cerebral blood flow. The mean source-detector distance (long-distance channels) was 38.1 ± 2.7 mm.

**Table 2:** fNIRS channel locations

Channel–fNIRS	10–20-system	MNI ccordinates	Landmark (% of overlap)	Hemisphere
S1-D1 / Ch1	FC3-FC1	− 38 12 55	Pre-Motor and Supplementary Motor Cortex (38%), DLPFC (35%), Frontal eye fields (23%)	LH
S1-D2 / Ch2	FC3-FC5	− 55 12 34	Pars opercularis (48%), Pre-Motor and Supplementary Motor Cortex (35%)	LH
S1-D3 / Ch3	FC3-C3	− 50–3 50	Pre-Motor and Supplementary Motor Cortex (62%), Primary Motor Cortex (18%)	LH
S2-D1 / Ch4	C1-FC1	− 26–5 68	Pre-Motor and Supplementary Motor Cortex (82%), Frontal eye fields (10%)	LH
S2-D3 / Ch5	C1-C3	− 42–20–62	Pars opercularis (48%), Pre-Motor and Supplementary Motor Cortex (35%)	LH
S2-D4 / Ch6	FC3-C3	− 50–3 50	Pre-Motor and Supplementary Motor Cortex (35%), Primary Motor Cortex (35%), Primary Somatosensory Cortex (35%)	LH
S3-D2 / Ch7	C5-FC5	− 62–3 23	Subcentral area (47%), Pre-Motor and Supplementary Motor Cortex (19%), Retrosubicular area (17%)	LH
S3-D3 / Ch8	C5-C3	− 60–18 37	Primary Somatosensory Cortex (41%), Subcentral area (21%)	LH
S4-D3 / Ch9	CP3-C3	− 52–34 52	Supramarginal gyrus (43%), Primary Somatosensory Cortex (43%)	LH
S4-D4 / Ch10	CP3-CP1	− 39–48 60	Supramarginal gyrus (42%), Somatosensory Association Cortex (27%), Primary Somatosensory Cortex (21%)	LH
S5-D5 / Ch11	FC4-C4	44 25 40	DLPFC (47%), Pars opercularis (27%), Pars triangularis Broca’s area (15%)	RH
S5-D6 / Ch12	FC4-C4	52–4 48	Pre-Motor and Supplementary Motor Cortex (57%), Primary Motor Cortex (19%), Primary Somatosensory Cortex (11%)	RH
S6-D5 / Ch13	C2-FC2	27–4 68	Primary Motor Cortex (37%), Primary Somatosensory Cortex (32%), Pre-Motor and Supplementary Motor Cortex (29%)	RH
S6-D6 / Ch14	C2-C4	42–21 62	Primary Motor Cortex (37%), Primary Somatosensory Cortex (32%), Pre-Motor and Supplementary Motor Cortex (29%)	RH
S6-D7 / Ch15	C2-CP2	28–36 71	Primary Somatosensory Cortex (38%), Primary Motor Cortex (24%), Pre-Motor and Supplementary Motor Cortex (18%), Somatosensory Association Cortex (17%)	RH
S7-D6 / Ch16	C6-C4	62–20 37	Primary Somatosensory Cortex (60%), Subcentral area (16%)	RH
S8-D6 / Ch17	CP4-C4	53–35 52	Supramarginal gyrus (50%), Primary Somatosensory Cortex (43%)	RH
S8-D7 / Ch18	CP4-CP2	39–49 60	Supramarginal gyrus (45%), Somatosensory Association Cortex (27%), Primary Somatosensory Cortex (22%)	RH

The fNIRS data was analyzed using the Satori (v.1.8) toolbox (Lührs and Goebel 2017). The onset times of hand movements were coded individually by applying NEUROGES and transcribed in the Satori toolbox. Triggers were set for postural control tasks. The raw intensity data were converted to optical density. Then optical density was converted via the modified Beer–Lambert law (MBLL) into concentration changes of oxygenated hemoglobin (∆HbO_2_) and deoxygenated hemoglobin (∆HbR). Movement artifacts were corrected by applying the motion correction functions of Satori (spike removal; temporal derivative distribution repair (TDDR) according to Fishburn et al. (2019)). Because the use of short-separation detector measurements as a regressor in the GLM has been previously shown to statistically improve HRF estimation (Gagnon et al. 2011; Yücel et al. 2015; Tachtsidis and Scholkmann 2016), we used short-distance signals to regress out signals from extra-cerebral layers from the long-distance channels. To account for cardiac oscillations and Mayer-waves we used a 0.4 Hz low-pass filter, a high-pass filter (butterworth) of 0.01 Hz, and the linear detrending function of Satori. The data was z-transformed, the betas of the hemodynamic response were estimated by a general linear model. Betas of each channel and condition were exported for further statistical analyses.

### Statistics

Comparisons of the mean(s) (repeated (rmANOVA) and univariate (uniANOVA) analyses of variance) were performed using IBM SPSS statistics (Version 28). Mean COP *length* was statistically analysed for the investigation of postural control (see Table 3). The exported betas of ∆HbO_2_ and ∆HbR were used for statistical analyses of brain activity (Table 4). For the betas as well as for COP *length* and *surface* we calculated the repeated within-subjects factors *vision* (eyes opened/closed), and *surface* (stable/unstable surface). For the analysis of brain activity we additionally integrated the within-subjects factor *channels* (ch; ch1—ch18). The between-subject factor *group* was calculated between blind and sighted individuals. Significant results are reported from p < 0.05. Multiple post hoc pairwise comparisons (18 tests for 18 channels) were corrected with Bonferroni corrections. If the requirements of the ANOVA (i.e., sphericity) were violated we used the Greenhouse–Geisser correction. We further analyzed by non-parametric Kruskal Wallis tests differences between the subgroups of congenitally blind individuals and individuals with acquired blindness.

**Table 3:** Statistical results of the postural control (center of pressure (COP)) of blind and sighted individuals (within-subjects conditions *vision* (open/closed eyes) and *surface* (stable/unstable surface))

COP length [mm/s]					
Factor (between groups)	F	df	p	partial η^2^	Pairwise comparison
Group * Vision	34.628	1, 18	< 0.01	0.658	Open, blind > sighted (p < 0.001, r = 0.73) Sighted, closed > open (p < 0.001, r = 0.73)
Group * Surface * Vision	44.560	1, 18	< 0.001	0.712	Stable, open, blind > sighted (p = 0.052, r = 0.44) Unstable, open, blind > sighted (p < 0.001, r = 0.79) Unstable, closed, sighted > blind (p < 0.05, r = 0.52) Sighted, stable, closed > open (p < 0.001, r = 0.86) Sighted, unstable, closed > open (p < 0.001, r = 0.97) Sighted, open, unstable > stable (p < 0.001, r = 0.98) Sighted, closed, unstable > stable (p < 0.001, r = 0.98) Blind, open, unstable > stable (p < 0.001, r = 0.97) Blind, closed, unstable > stable (p < 0.001, r = 0.99)

Factor (within groups)	F	df	p	partial η^2^	Pairwise comparison
Surface	676.496	1, 18	< 0.001	0.974	Unstable > stable (p < 0.001, r = 0.98)
Vision	50.928	1, 18	< 0.001	0.739	Closed > open (p < 0.001, r = 0.70)
Surface * Vision	73.018	1, 18	< 0.001	0.802	Stable, closed > opened (p < 0.01, r = 0.50) Unstable, closed > open (p < 0.001, r = 0.71) Open, unstable > stable (p < 0.001, r = 0.94) Closed, unstable > stable (p < 0.001, r = 0.96)

**Table 4:** Statistical fNIRS results (∆HbO_2_/∆HbR) for blind and sighted individuals (within-subjects conditions *vision* (open/closed eyes) and *surface* (stable/unstable surface))

∆HbO_2_							
Factor (between groups)	F		df		p	Partial η^2^	Pairwise comparison
Group							
Ch6	6.957		1, 18		< 0.05	0.279	Blind > sighted (p < 0.05, r = 0.54)
Ch16	5.071		1, 18		< 0.05	0.220	Blind > sighted (p < 0.05, r = 0.45)
Ch18	3.861		1, 18		= 0.065	0.177	Blind > sighted (p = 0.065, r = 0.42)

Factor (within groups)	F		df		p	partial η^2^	Pairwise comparison
Surface Ch10	4.031		1, 18		= 0.060	0.183	Unstable > stable (p = 0.060, r = 0.73)
Surface * Vision Ch18	6.088		1, 18		< 0.05	0.253	Closed eyes, unstable surface > stable surface (p = 0.065, r = 0.06) Stable surface, open > closed (p < 0.05, r = 0.52)

∆HbR							
Factor (between groups)		F		df	p	partial η^2^	Pairwise comparison
Group * vision Ch1		6.345		1, 18	< 0.05	0.261	Sighted, closed < open (p < 0.05, r = 0.67)
Ch11		7.564		1, 18	< 0.05	0.296	Sighted, closed < open (p < 0.05, r = 0.51)
Group * surface Ch6		4.322		1, 18	= 0.052	0.194	Stable, sighted < blind (p < 0.05, r = 0.46) Blind, unstable < stable (p < 0.05, r = 0.67)

Factor (within groups)		F		df	p	partial η^2^	Pairwise comparison
Vision Ch4		6.178		1, 18	< 0.05	0.256	Closed < open (p < 0.05, r = 0.52)
Ch8		4.072		1, 18	= 0.059	0.184	Closed < open (p = 0.06, r = 0.45)
Ch9		4.266		1, 18	= 0.054	0.192	Closed < open (p = 0.05, r = 0.42)
Ch13		5.847		1, 18	< 0.05	0.245	Closed < open (p < 0.05, r = 0.45)
Ch14		5.333		1, 18	< 0.05	0.229	Closed < open (p < 0.05, r = 0.41)

## Results

### Postural Control

#### ANOVA (Between Groups)

**Fig. 2:**
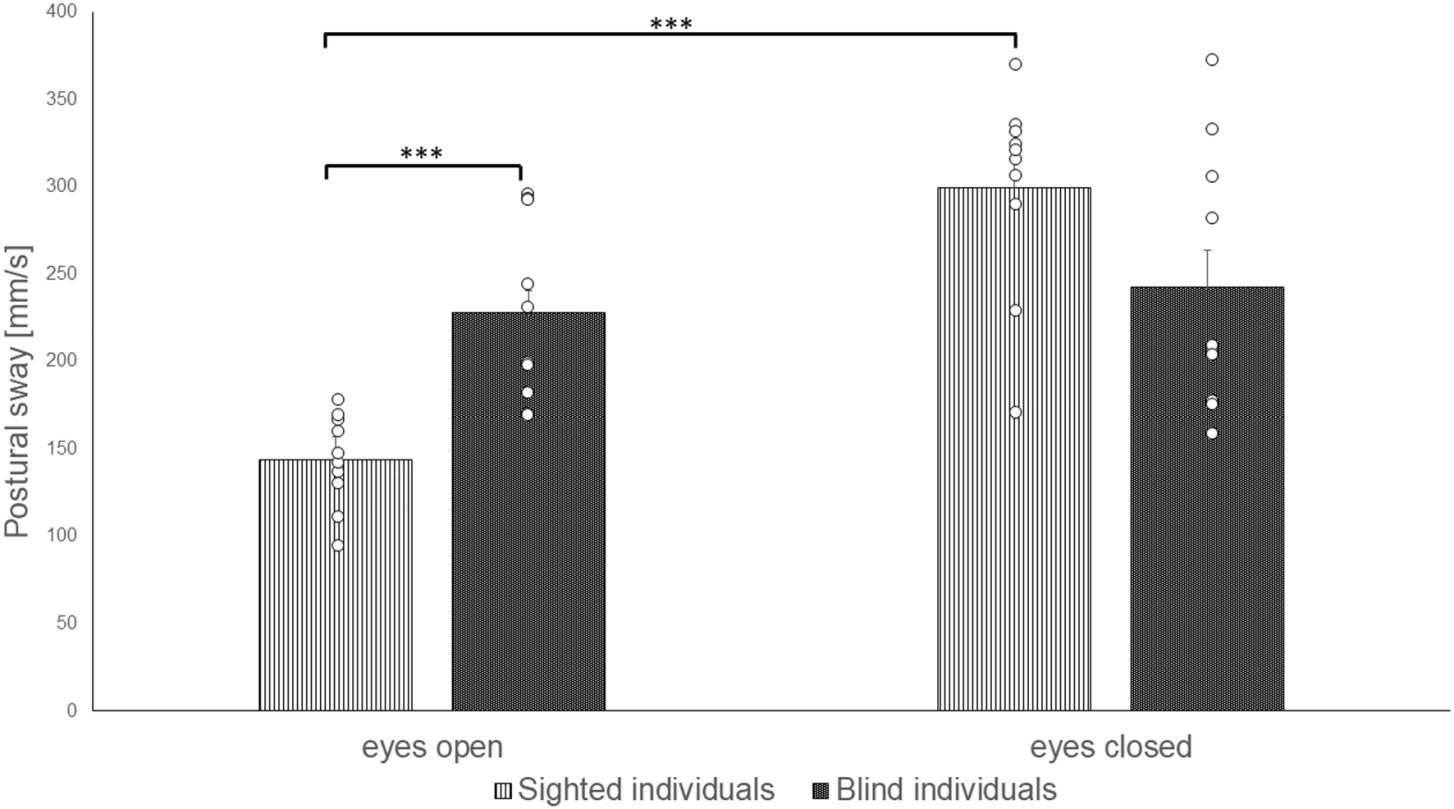
Postural sway (length of the center of pressure per second [mm/s]) of blind and sighted individual during conditions with opened and closed eyes

The rmANOVA of the COP *length* [mm/s] values revealed statistical significant effects between groups for the factors group * vision (F(1, 18) = 34.628, p < 0.001, eta square [η^2^] = 0.658), and group * vision * surface (F(1, 18) = 44.560, p < 0.001, η^2^ = 0.712; Table 3). Post-hoc comparisons for the interaction of group * vision revealed significantly increased postural sway for blind individuals when compared to the sighted individuals during the eyes opened condition (p < 0.001, (effect size Cohen’s) r = 0.73). The postural sway of sighted individuals (but not blind individuals) was significantly increased during the eyes closed when compared to the eyes open condition (p < 0.001, r = 0.97; Fig. 2).

**Fig. 3:**
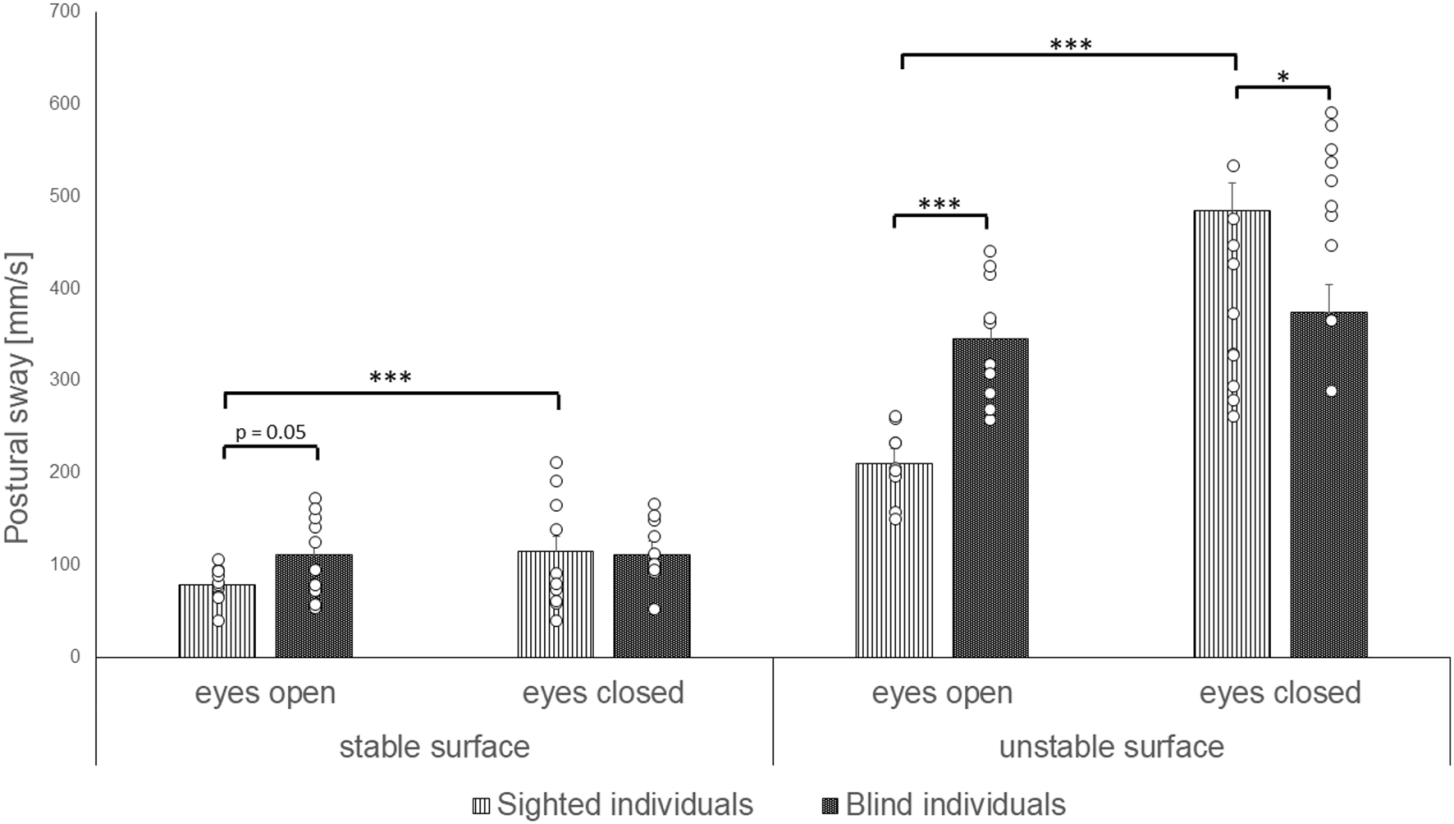
Postural sway (length of the center of pressure per second [mm/s]) of blind and sighted individual during conditions with opened and closed eyes and the stable and unstable surfaces

Post-hoc comparisons for the factors group * vision * surface revealed significantly increased postural sway for blind individuals when compared to the sighted individuals during the eyes open condition and when standing on the stable (p = 0.052, r = 0.44) as well as on the unstable surface (p < 0.001, r = 0.79). Sighted individuals showed an increased postural sway when compared to blind individuals during the eyes closed and unstable surface condition (p < 0.05, r = 0.52). The postural sway of the sighted group was significantly increased during the eyes closed condition when compared to the eyes open condition and when standing on the stable (p < 0.001, r = 0.86) as well as on the unstable surface (p < 0.001, r = 0.97). Blind and sighted individuals showed increased postural sway during the unstable surface condition when compared to the stable surface condition with open eyes as (p < 0.001, r_sighted_ = 0.98, r_blind_ = 0.98) well as with closed eyes (p < 0.001, r_sighted_ = 0.97, r_blind_ = 0.99; Fig. 3).

### Sub-Group Analysis (Between Blind Groups)

A Kruskal Wallis test between the subgroups of congenitally blind individuals and individuals with acquired blindness did not reveal significant differences.

### ANOVA (Within Groups)

Within groups effects were found for *surface* (F(1, 18) = 676.496, p < 0.001, η^2^ = 0.974), *vision* (F(1, 18) = 50.928, p < 0.001, η^2^ = 0.739), and vision * surface (F(1, 18) = 73.018, p < 0.001, η^2^ = 0.802). Post-hoc comparisons for the factor *surface* revealed significantly increased postural sway during the unstable surface condition when compared to the stable surface condition (p < 0.001, r = 0.98). Post-hoc comparisons for the factor *vision* revealed significantly increased postural sway during the eyes closed condition when compared to the condition with open eyes (p < 0.001, r = 0.70). Post-hoc comparisons for the interaction effect of vision * surface revealed significantly increased postural sway during the eyes closed condition when compared to the open eyes condition during the stable surface condition (p < 0.05, r = 0.50) and during the unstable surface condition (p < 0.001, r = 0.71). Furthermore, postural sway significantly increased during the open eyes (p < 0.001, r = 0.94) and closed eyes condition (p < 0.001, r = 0.96) during the unstable surface condition when compared to the stable surface condition.

### Brain Oxygenation

#### ANOVA (Between Groups)

**Fig. 4:**
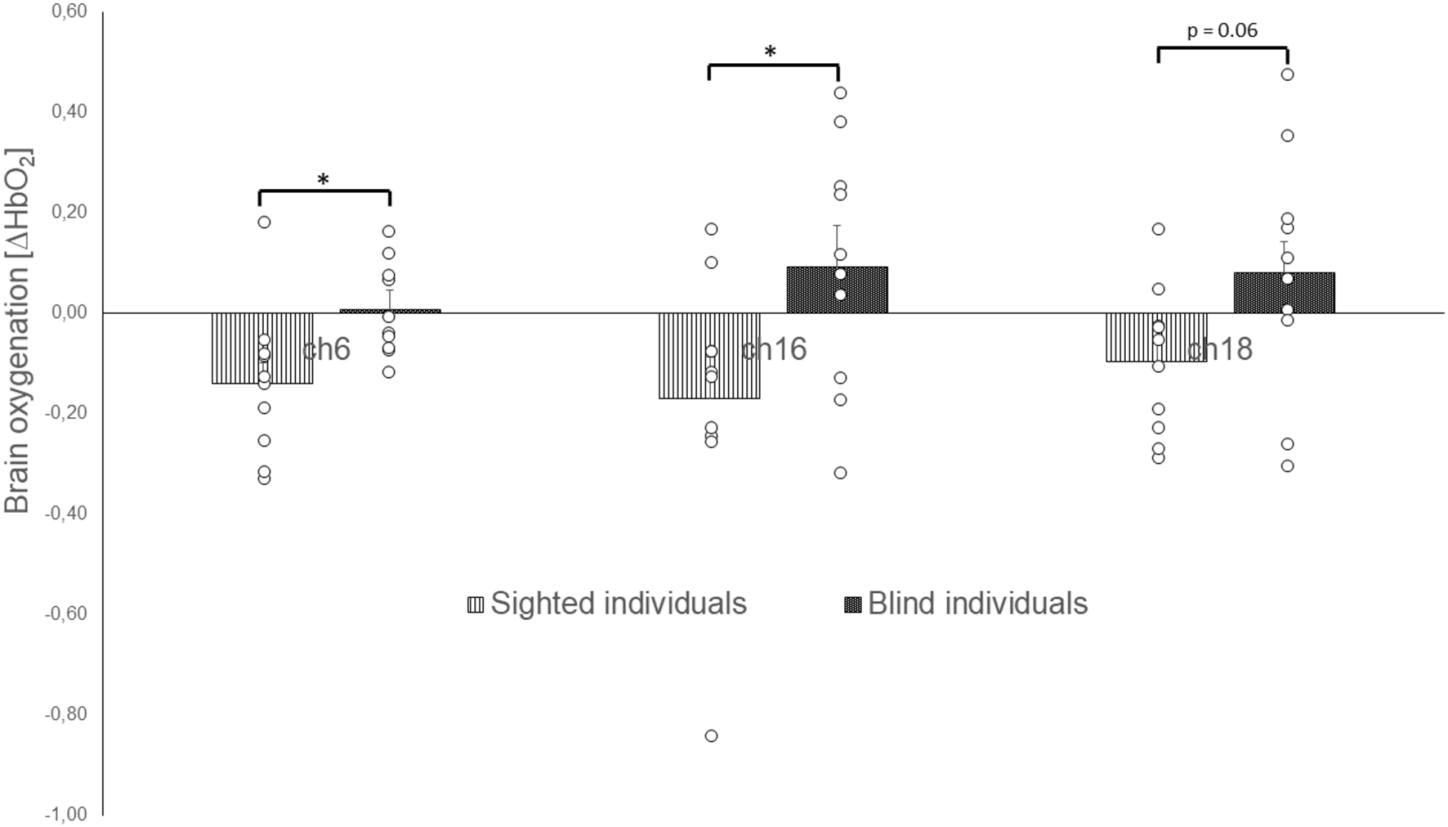
Brain oxygenation (∆HbO_2_) of blind and sighted individuals during postural control

The uniANOVA for the ∆HbO_2_ values revealed significant effects for the factor *group* in channel 6 (ch6; F(1, 18) = 6.957, p < 0.05, η^2^ = 0.279)), ch16(F(1, 18) = 5.071, p < 0.05, η^2^ = 0.220), and ch18(F(1, 18) = 3.861, p = 0.065, η^2^ = 0.177; Table 4). Post-hoc comparisons showed that was significantly increased ∆HbO_2_ in blind individuals when compared to sighted individuals for ch6 (p < 0.05, r = 0.54), ch16 (p < 0.05, r = 0.45), and ch18 (p = 0.065, r = 0.42; Fig. 4).

**Fig. 5:**
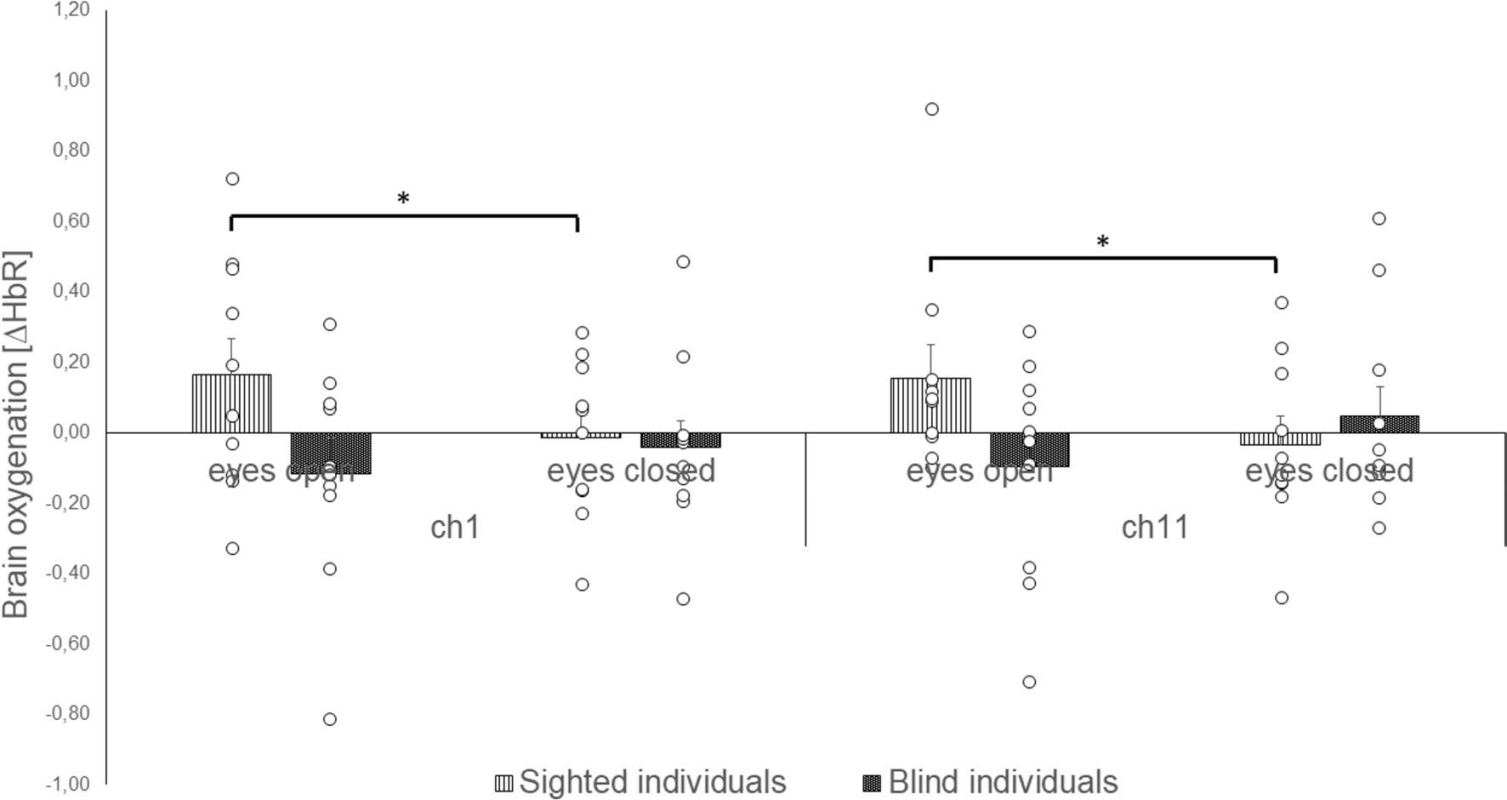
Brain oxygenation (∆HbO_2_) of blind and sighted individuals during postural control with open and closed eyes

The uniANOVA for the ∆HbR values revealed a significant effect for the interaction of *group* * *vision* in ch1(F(1, 18) = 6.345, p < 0.05, η^2^ = 0.261), and ch11(F(1, 18) = 7.564, p < 0.05, η^2^ = 0.296). Post-hoc comparisons showed in ch1 (p < 0.05, r = 0.67) as well as in ch11 (p < 0.05, r = 0.51) significantly reduced ∆HbR in sighted individuals during the eyes closed condition when compared to the eyes open condition (Fig. 5).

The uniANOVA for the ∆HbR values further revealed a marginally significant effect for the interaction of *group* * *surface* in ch6(F(1, 18) = 4.322, p = 0.052, η^2^ = 0.194). Post-hoc comparisons showed significantly reduced ∆HbR in sighted when compared to blind individuals during the stable surface condition (p < 0.05, r = 0.46). Furthermore, blind individuals showed significantly reduced ∆HbR during the unstable when compared to the stable surface condition (p < 0.05, r = 0.67).

#### Sub-Group Analysis (Between Blind Groups)

A Kruskal Wallis test between the subgroups of congenitally blind individuals and individuals with acquired blindness for the ∆HbO_2_ values revealed significant differences during unstable surface conditions (with eyes closed) in ch16(H(1) = 5.500, p < 0.05) showing increased ∆HbO_2_ in individuals with acquired blindness. For the ∆HbR, significant differences were observed (also during the unstable surface condition with closed eyes) in ch11(H(1) = 6.545, p < 0.05) by reduced ∆HbR in congenitally blind individuals.

#### ANOVA (Within Groups)

Independent from effects between groups, the uniANOVA revealed marginally significant effects for the ∆HbO_2_ values and the factor *surface* in ch10(F(1, 18) = 4.031, p = 0.060, η^2^ = 0.183; Table 4). Post-hoc comparisons showed that ∆HbO_2_ significantly increased during the unstable surface condition when compared to stable surface condition (p = 0.060, r = 0.42). The uniANOVA further revealed significant interaction effects of *surface* and *vision* for the ∆HbO_2_ values in ch18(F(1, 18) = 6.088, p < 0.05, η^2^ = 0.253). Post-hoc comparisons showed that ∆HbO_2_ (marginally) significantly increased during the unstable surface condition with closed eyes and when compared to the stable condition (p = 0.065, r = 0.06). Furthermore, the post-hoc comparison showed significantly increased oxygenation during eyes open when compared to eyes closed during the stable surface condition (p < 0.05, r = 0.52).

The uniANOVA revealed for the ∆HbR values significant effects of *vision* in ch4(F(1, 18) = 6.178, p < 0.05, η^2^ = 0.253), ch8(F(1, 18) = 4.072, p = 0.059, η^2^ = 0.184), ch9(F(1, 18) = 4.266, p = 0.054, η^2^ = 0.192), ch13(F(1, 18) = 5.847, p < 0.05, η^2^ = 0.245), and ch14(F(1, 18) = 5.333, p < 0.05, η^2^ = 0.229). Post-hoc comparisons showed in all channels that ∆HbR significantly decreased during the eyes closed condition when compared to the open eyes condition (ch4, p < 0.05, r = 0.52; ch8, p = 0.059, r = 0.45; ch9, p = 0.054, r = 0.42; ch13, p < 0.05, r = 0.45; ch14, p < 0.05, r = 0.41).

## Discussion

The present study compared neuronal control of posture in blind and sighted individuals within sensorimotor cortices. The analysis of postural sway revealed that blind individuals are characterized by increased postural sway when compared to the sighted individuals during eyes opened conditions (on the stable and on the unstable surface). However, blind individuals show less postural sway (/better balance performances) than sighted individuals during the eyes closed condition and unstable surface. Sighted individuals (but not blind individuals) instead show increased postural sway during the closed eyes condition and when compared to the open eyes condition (on the stable and on the unstable surface). Both groups showed increased postural sway during the unstable surface condition when compared to the condition with the stable surface as well as during the eyes closed condition when compared to the condition with open eyes.

The analysis of the brain oxygenation revealed that blind individuals are characterized by increased brain oxygenation overall conditions when compared to sighted individuals. Blind individuals showed increased neuronal activity during the stable and unstable surface condition with open eyes when compared to the sighted group. Sighted individuals showed increased brain activation during the closed eyes condition when compared to the open eyes condition as well as during the closed eyes condition on the unstable surface when compared to the eyes open condition. Both groups presented increased brain activation during the eyes closed condition when compared to eyes open condition as well as during the unstable surface condition when compared to stable surface condition.

### Group Effects

The present study revealed that blind individuals control posture based on altered neuronal mechanisms within the sensorimotor system when compared to sighted individuals. In fact, when compared to sighted individuals and overall conditions, the blind individuals showed increased brain activity in two channels covering frontal brain cortices of the left (LH) and right hemisphere (RH), i.e., the inferior opercularis (LH) and the frontal middle cortex (RH). Based on the fact that congenitally blind individuals are characterized by decreased postural stability when compared to sighted individuals but more stable when compared to sighted but blind-folded individuals (Ribadi et al. 1987), it has been hypothesized that blind individuals would control posture by different neuronal mechanisms than sighted individuals (Ribadi et al. 1987). Ribadi et al (1987) hypothesized that congenitally blind individuals may adapt neuronally in order to control posture by feedforward processes (/anticipatory balance control) rather than by feedback control. Furthermore, the investigation of automatic postural responses to platform displacements during postural control did not result in different EMG latencies of the lower extremity muscles in response to perturbations between sighted and blind individuals (Nakata and Yabe 2001). However, blind individuals presented faster reaction times to somatosensory stimuli triggered by platform displacements measured by pushing a hand-held button (Nakata and Yabe 2001). Nakata and Yabe (2001) concluded that blindness may not affect the spinal stretch reflex, but may affect a volitional act mediated through the motor cortex. In fact, previous studies applying fNIRS during postural control tasks revealed that increased activation in the dorsolateral prefrontal cortex (DLPFC) may be indicative of increased attentional resources being used to maintain balance (Teo et al. 2018). Corbetta and Shulman (2002) hypothesized that it exists a neuroanatomical model of attentional control of frontoparietal brain areas that link relevant sensory representations to relevant motor representations (Corbetta and Shulman 2002). These results may explain the present results of increased brain activation in blind individuals as they may increase their attention to other sensory modalities to control posture without visual information. In order to perform postural control by feedforward mechanisms of focusing the attention towards additional sensory stimuli (other than visual information) blind individuals may have adapted in the sensorimotor cortex to control balance by increased activation patterns during its execution. The frontal cortex of the right hemisphere may be particularly involved in the cognitive selection of sensory information and responses by feedforward/top-down control of attention (Corbetta and Shulman 2002). In blind individuals, visual and multisensory brain regions do not receive their expected input forcing them to use their remaining senses to cope with everyday demands for which the deprived sense might have been instrumental (Roeder et al. 2021). Blind individuals are therefore characterized by intramodal neuroplasticity (Röder and Neville 2003). It has been argued that development of use-dependent cortical reorganization may be a consequence of the absence of visual input (Elbert et al. 2002). Thus, blind individuals may reorganize / increase their somatosensory attention to control posture in the absence of visual input.

Furthermore, blind individuals were particularly characterized by increased postural sway and increased brain oxygenation during the balance condition with open eyes. This strengthens the fact that blind but not sighted individuals must integrate other sensory modalities during postural control, particularly when balancing with “open eyes”. In contrast to blind individuals, sighted individuals increased their brain oxygenation in sensorimotor cortices during the most complex condition, i.e., when controlling posture with closed eyes and standing on the unstable surface. We therefore conclude that the sensorimotor cortex of blind individuals adapts (intramodal) in order to control posture based on the increased attention to other sensory stimuli than vision. Sighted individuals adapt to increased sensory demands during postural control by increasing their brain oxygenation in sensorimotor cortices during most balance situations without vision on unstable surfaces.

### Within-Subjects Effects

Overall groups, the data showed that postural sway increases during a condition with closed eyes when compared to a condition with open eyes. However, knowing the group effects (i.e., no changes of blind individuals between open and closed eyes conditions), this result is rather grounded in the increased postural sway of sighted individuals from eyes open to eyes closed conditions. With regards to brain activation, the sensorimotor cortex also shows increased oxygenation patterns in several channels. Thus, it seems that the sensorimotor cortex is increasingly activated when balancing with closed eyes when compared to open eyes. This result also strengthens the assumption that individuals recruit additional resources to control posture without visual information. Thus, sighted individuals recruit additional neuronal sensorimotor structures for the balance control without vision, i.e., during balance situations that are characterized by increased instability. Previously, activity in the superior frontal gyrus showed to be a representative of decreased postural control (Lehmann et al. 2022). St George et al. (2021) also showed in the prefrontal cortex that neural activity increased with increasing balance difficulty. The investigation of spontaneous brain activity between eyes open and eyes closed resting states revealed that brain activity in the eyes open condition is significantly greater in attentional system areas, including the fusiform gyrus, occipital and parietal cortex, but significantly lower in sensorimotor system areas, including the precentral/postcentral gyrus, paracentral lobule (PCL) and temporal cortex compared to the eyes closed condition (Wei et al. 2018). The present results therefore indicate that postural control during eyes closed conditions increases the activation in sensorimotor areas to compensate for balancing without vision.

Furthermore, postural sway also increased during the unstable surface condition when compared to the stable surface (with open eyes as well as with closed eyes). Brain oxygenation also increased with unstable surface conditions when compared to the stable surface condition. The investigation of temporo-parietal brain areas during balance control with sensory manipulations (e.g., eyes open in the dark and sway referenced floor) revealed increased bilateral activation in the superior temporal gyrus, STG, and supramarginal gyrus, SMG when both vision and proprioceptive information were degraded (Karim et al. 2013). The authors concluded that those postural situations force individuals to rely on primarily vestibular information in the control of balance (Karim et al. 2013). Our data further indicates that the sensorimotor system additionally adds relevant neural information to control posture without visual information and decreased underground stability. We therefore conclude that all individuals, blind and sighted, increase their brain activation in sensorimotor cortices during balance situations without visual information and reduced stability from the surface in order to compensate for reduced sensory input from the visual modality.

### Limitations and Conclusion

Although the present data provides new insights about postural control in sighted and blind individuals some limitations of the study must be considered. First, blind and sighted individuals concerned active athletes. In contrast to the general blind population such individuals are very mobile in their daily life. Thus, the data may not be generalized overall blind individuals. In fact, blind athletes showed to be more stable than blind non-athletes (Aydoǧ et al. 2006). Furthermore, the blind population of the present study was characterized by a sample of congenitally blind individuals and individuals with aquired blindness during life. It has been described that such populations may adapt differently to visual deprivation (Roeder et al. 2021). We therefore further analyzed these two subgroups. We did not identify differences on postural control but minor differences in their brain oxygenation patterns. However, because of the limited sample size this should be investigated in a bigger sample of individuals because the present findings constitute only minor differences. Thirdly, the fNIRS system that was applied in the present study is limited regarding its spatial distribution. Thus, other brain regions may be critically involved that were not covered by the present fNIRS system. Still, the data showed for the first time that sighted individuals increase their brain activation in sensorimotor cortices during altered sensory integration during postural control. Blind individuals are characterized by increased neuronal activity overall conditions indicating additional sensory integration without vision during postural control when compared to sighted individuals. Thus, the sensorimotor cortex of blind individuals adapts to control posture without vision. Because blind individuals have been characterized by intramodal plasticity, we conclude that these processes represent adaptations of the sensorimotor cortex of blind individuals to control posture without vision.

## Supplementary Information

Below is the link to the electronic supplementary material.

Supplementary file1 (DOCX 17 KB)

## Data Availability

The data will be shared upon request.

## References

[CR1] Aydoǧ E, Aydoǧ ST, Çakci A, Doral MN (2006) Dynamic postural stability in blind athletes using the biodex stability system. Int J Sports Med 27:415–418. 10.1055/S-2005-865777 16729385 10.1055/S-2005-865777

[CR2] Basso Moro S, Bisconti S, Muthalib M et al (2014) A semi-immersive virtual reality incremental swing balance task activates prefrontal cortex: a functional near-infrared spectroscopy study. Neuroimage 85:451–460. 10.1016/j.neuroimage.2013.05.031 23684867 10.1016/j.neuroimage.2013.05.031

[CR3] Choy NL, Brauer S, Nitz J (2003) Changes in postural stability in women aged 20 to 80 years. J Gerontol A Biol Sci Med Sci 58:525–530. 10.1093/GERONA/58.6.M525 12807923 10.1093/GERONA/58.6.M525

[CR4] Corbetta M, Shulman GL (2002) Control of goal-directed and stimulus-driven attention in the brain. Nat Rev Neurosci 33(3):201–215. 10.1038/nrn755 10.1038/nrn755 11994752

[CR5] Elbert T, Sterr A, Rockstroh B et al (2002) Expansion of the tonotopic area in the auditory cortex of the blind. J Neurosci 22:9941–9944. 10.1523/JNEUROSCI.22-22-09941.2002 12427851 10.1523/JNEUROSCI.22-22-09941.2002 PMC6757838

[CR6] Ferrari M, Bisconti S, Spezialetti M et al (2014) Prefrontal cortex activated bilaterally by a tilt board balance task: A functional near-infrared spectroscopy study in a semi-immersive virtual reality environment. Brain Topogr. 10.1007/s10548-013-0320-z 24101293 10.1007/s10548-013-0320-z

[CR7] Fishburn FA, Ludlum RS, Vaidya CJ, Medvedev AV (2019) Temporal derivative distribution repair (TDDR): a motion correction method for fNIRS. Neuroimage 184:171–179. 10.1016/J.NEUROIMAGE.2018.09.025 30217544 10.1016/J.NEUROIMAGE.2018.09.025 PMC6230489

[CR8] Gagnon L, Perdue K, Greve D et al (2011) Improved recovery of the hemodynamic response in diffuse optical imaging using short optode separations and state-space modeling. Neuroimage 56:1362–1371. 10.1016/J.NEUROIMAGE.2011.03.001 21385616 10.1016/J.NEUROIMAGE.2011.03.001 PMC3085546

[CR9] Giagazoglou P, Amiridis IG, Zafeiridis A et al (2009) Static balance control and lower limb strength in blind and sighted women. Eur J Appl Physiol 107:571–579. 10.1007/S00421-009-1163-X 19701648 10.1007/S00421-009-1163-X

[CR10] Helmich I, Berger A, Lausberg H (2016) Neural control of posture in individuals with persisting postconcussion symptoms. Med Sci Sports Exerc 48:2362–2369. 10.1249/MSS.0000000000001028 27387294 10.1249/MSS.0000000000001028

[CR11] Helmich I, Coenen J, Henckert S et al (2020) Reduced frontopolar brain activation characterizes concussed athletes with balance deficits. NeuroImage Clin 25:102164. 10.1016/j.nicl.2020.102164 31954336 10.1016/j.nicl.2020.102164 PMC6965737

[CR12] Herold F, Orlowski K, Börmel S, Müller NG (2017) Cortical activation during balancing on a balance board. Hum Mov Sci. 10.1016/j.humov.2016.11.002 27846398 10.1016/j.humov.2016.11.002

[CR13] Horak FB (2006) Postural orientation and equilibrium: What do we need to know about neural control of balance to prevent falls? Age Ageing 35(Suppl2):ii7–ii11 16926210 10.1093/ageing/afl077

[CR14] Johansson J, Nordström A, Gustafson Y et al (2017) Increased postural sway during quiet stance as a risk factor for prospective falls in community-dwelling elderly individuals. Age Ageing 46:964–970. 10.1093/AGEING/AFX083 28531243 10.1093/AGEING/AFX083

[CR15] Karim H, Schmidt B, Dart D et al (2012) Functional near-infrared spectroscopy (fNIRS) of brain function during active balancing using a video game system. Gait Posture. 10.1016/j.gaitpost.2011.10.007 22078300 10.1016/j.gaitpost.2011.10.007 PMC3294084

[CR16] Karim H, Fuhrman SI, Sparto P et al (2013) Functional brain imaging of multi-sensory vestibular processing during computerized dynamic posturography using near-infrared spectroscopy. Neuroimage 74:318–325. 10.1016/j.neuroimage.2013.02.010 23419940 10.1016/j.neuroimage.2013.02.010 PMC3677521

[CR17] Lehmann N, Kuhn YA, Keller M et al (2022) Brain activation during active balancing and its behavioral relevance in younger and older adults: a functional near-infrared spectroscopy (fNIRS) study. Front Aging Neurosci. 10.3389/FNAGI.2022.828474/FULL 35418854 10.3389/FNAGI.2022.828474/FULL PMC8997341

[CR18] Lin CC, Barker JW, Sparto PJ et al (2017) Functional near-infrared spectroscopy (fNIRS) brain imaging of multi-sensory integration during computerized dynamic posturography in middle-aged and older adults. Exp Brain Res. 10.1007/s00221-017-4893-8 28197672 10.1007/s00221-017-4893-8 PMC5494712

[CR19] Lührs M, Goebel R (2017) Turbo-Satori: a neurofeedback and brain-computer interface toolbox for real-time functional near-infrared spectroscopy. Neurophotonics 4:1. 10.1117/1.NPH.4.4.041504 10.1117/1.NPH.4.4.041504 PMC5629919 29021985

[CR20] Mihara M, Miyai I, Hatakenaka M et al (2008) Role of the prefrontal cortex in human balance control. Neuroimage. 10.1016/j.neuroimage.2008.07.029 18718542 10.1016/j.neuroimage.2008.07.029

[CR21] Nakata H, Yabe K (2001) Automatic postural response systems in individuals with congenital total blindness. Gait Posture 14:36–43. 10.1016/S0966-6362(00)00100-4 11378423 10.1016/S0966-6362(00)00100-4

[CR22] Ozdemir RA, Pourmoghaddam A, Paloski WH (2013) Sensorimotor posture control in the blind: superior ankle proprioceptive acuity does not compensate for vision loss. Gait Posture 38:603–608. 10.1016/J.GAITPOST.2013.02.003 23477840 10.1016/J.GAITPOST.2013.02.003

[CR23] Ribadi H, Rider RA, Toole T (1987) A Comparison of Static and Dynamic Balance in Congenitally Blind, Sighted, and Sighted Blindfolded Adolescents. Adapt Phys Act Q 4:220–225. 10.1123/apaq.4.3.220 10.1123/apaq.4.3.220

[CR24] Röder B, Neville H (2003) Developmental functional plasticity. In: Robertson I (ed) Grafman J, I. Handbook of Neuropsychology. Elsevier, Amsterdam

[CR25] Röder B, Teder-Sälejärvi W, Sterr A et al (1999) Improved auditory spatial tuning in blind humans. Nature 400:162–166. 10.1038/22106 10408442 10.1038/22106

[CR26] Roeder B, Kekunnaya R, Guerreiro MJS (2021) Neural mechanisms of visual sensitive periods in humans. Neurosci Biobehav Rev 120:86–99. 10.1016/J.NEUBIOREV.2020.10.030 33242562 10.1016/J.NEUBIOREV.2020.10.030

[CR27] Rougier P, Farenc I (2000) Adaptative effects of loss of vision on upright undisturbed stance. Brain Res 871:165–174. 10.1016/S0006-8993(00)02357-X 10899283 10.1016/S0006-8993(00)02357-X

[CR28] Scholkmann F, Kleiser S, Metz AJ et al (2014) A review on continuous wave functional near-infrared spectroscopy and imaging instrumentation and methodology. Neuroimage 85(Pt 1):6–27. 10.1016/j.neuroimage.2013.05.004 23684868 10.1016/j.neuroimage.2013.05.004

[CR29] Schwesig R, Goldich Y, Hahn A et al (2011) Postural control in subjects with visual impairment. Eur J Ophthalmol 21:303–309. 10.5301/EJO.2010.5504 20853264 10.5301/EJO.2010.5504

[CR30] Sobry V, Badin P, Cernaianu S et al (2014) Do visually impaired people have a static balance as effective as sighted people? NeuroRehabilitation 35:851–861. 10.3233/NRE-141181 25361555 10.3233/NRE-141181

[CR31] St George RJ, Hinder MR, Puri R et al (2021) Functional near-infrared spectroscopy reveals the compensatory potential of pre-frontal cortical activity for standing balance in young and older adults. Neuroscience 452:208–218. 10.1016/J.NEUROSCIENCE.2020.10.027 33197501 10.1016/J.NEUROSCIENCE.2020.10.027

[CR32] Stel VS, Smit JH, Pluijm SMF, Lips P (2004) Consequences of falling in older men and women and risk factors for health service use and functional decline. Age Ageing 33:58–65. 10.1093/AGEING/AFH028 14695865 10.1093/AGEING/AFH028

[CR33] Tachtsidis I, Scholkmann F (2016) False positives and false negatives in functional near-infrared spectroscopy: issues, challenges, and the way forward. Neurophotonics. 10.1117/1.NPh.3.3.031405 27054143 10.1117/1.NPh.3.3.031405 PMC4791590

[CR34] Teo WP, Goodwill AM, Hendy AM et al (2018) Sensory manipulation results in increased dorsolateral prefrontal cortex activation during static postural balance in sedentary older adults: An fNIRS study. Brain Behav 8:1109. 10.1002/BRB3.1109 10.1002/BRB3.1109 PMC6192391 30230687

[CR35] Wei J, Chen T, Li C et al (2018) Eyes-open and eyes-closed resting states with opposite brain activity in sensorimotor and occipital regions: Multidimensional evidences from machine learning perspective. Front Hum Neurosci 12:422. 10.3389/FNHUM.2018.00422/BIBTEX 30405376 10.3389/FNHUM.2018.00422/BIBTEX PMC6200849

[CR36] Wolinsky FD, Fitzgerald JF, Stump TE (1997) The effect of hip fracture on mortality, hospitalization, and functional status: a prospective study. Am J Public Health 87:398. 10.2105/AJPH.87.3.398 9096540 10.2105/AJPH.87.3.398 PMC1381011

[CR37] Yücel M, Selb J, Aasted C et al (2015) Short separation regression improves statistical significance and better localizes the hemodynamic response obtained by near-infrared spectroscopy for tasks with differing autonomic responses. Neurophotonics 2:035005. 10.1117/1.NPH.2.3.035005 26835480 10.1117/1.NPH.2.3.035005 PMC4717232

