# Supplementary material for: Neuronal Control of Posture in Blind Individuals
Source: Brain Topogr. 2024 Mar 15;37(5):783–95. doi: 10.1007/s10548-024-01041-7 (PMC11393032; doi:10.1007/s10548-024-01041-7)
Supplement: Supplementary file 1 — Supplementary file1 (DOCX 17 KB) [file 10548_2024_1041_MOESM1_ESM.docx]

**Legends**

**Figures**

**Figure 1.** (A) Exemplary measurement / participant; (B) fNIRS optode placement according to the 10-20-system; (C) fNIRS topographical layout; (D) fNIRS sensitivity map; (E) block design (6 trials per condition separated in 2 blocks).

**Figure 2.** Postural sway (length of the center of pressure per second [mm/s]) of blind and sighted individual during conditions with opened and closed eyes.

**Figure 3.** Postural sway (length of the center of pressure per second [mm/s]) of blind and sighted individual during conditions with opened and closed eyes and the stable and unstable surfaces.

**Figure 4.** *Brain oxygenation* (∆HbO_2_) of blind and sighted individuals during postural control.

**Figure 5.** *Brain oxygenation* (∆HbR) of blind and sighted individuals during postural control with open and closed eyes.

**Tables**

**Table 01.** Participants.

**Table 02.** fNIRS channel locations.

**Table 03.** Statistical results of the postural control (center of pressure (COP)) of blind and sighted individuals (within-subjects conditions *vision* (open/closed eyes) and *surface* (stable/unstable surface)).

**Table 04.** Statistical fNIRS results (∆HbO_2_/∆HbR) for blind and sighted individuals (within-subjects conditions *vision* (open/closed eyes) and *surface* (stable/unstable surface)).
